# An Apoptotic and Endosymbiotic Explanation of the Warburg and the Inverse Warburg Hypotheses

**DOI:** 10.3390/ijms19103100

**Published:** 2018-10-10

**Authors:** Szymon Kaczanowski, Joanna Klim, Urszula Zielenkiewicz

**Affiliations:** Institute of Biochemistry and Biophysics, Polish Academy of Sciences, Pawińskiego 5a Str., 02-106 Warsaw, Poland; klim@ibb.waw.pl (J.K.); ulazet@ibb.waw.pl (U.Z.)

**Keywords:** apoptosis, Warburg effect, inverse Warburg effect, yeast, Alzheimer

## Abstract

Otto Warburg, a Nobel prize winner, observed that cancer cells typically “switch” from aerobic to anaerobic respiration. He hypothesized that mitochondrial damage induces neoplastic transformation. In contrast, pathological aging is observed mainly in neuron cells in neurodegenerative diseases. Oxidative respiration is particularly active in neurons. There is inverse comorbidity between cancer and neurodegenerative diseases. This led to the creation of the “inverse Warburg hypothesis”, according to which excessive mitochondrial activity induces pathological aging. The findings of our studies suggest that both the Warburg effect and the “inverse Warburg hypothesis” can be elucidated by the activation or suppression of apoptosis through oxidative respiration. The key outcome of our phylogenetic studies was the discovery that apoptosis and apoptosis-like cell death evolved due to an evolutionary “arms race” conducted between “prey” protomitochondrion and “predator” primitive eukaryotes. The ancestral protomitochondrial machinery produces and releases toxic mitochondrial proteins. Extant apoptotic factors evolved from these toxins. Our experiments indicate that the mitochondrial machinery is directly involved in adaptation to aerobic conditions. Additionally, our hypothesis is supported by the fact that different apoptotic factors are directly involved in respiration.

## 1. Introduction

Aging is one of the biggest mysteries of human biology. The progress of organismal aging leads inevitably to age-related diseases, such as cancer and neurodegenerative disease [1,2,3]. However, the mechanisms involved in cancer formation are almost the reverse of those involved in neurodegenerative diseases.

The development of both diseases is caused by perturbations in apoptosis regulation, which is a type of programmed cell death. Apoptosis can be distinguished from other kinds of cell death. There are cytological hallmarks of apoptosis such as the release of mitochondrial apoptotic factors, activation of apoptotic proteases, disintegration of the nucleus, and self-degradation of DNA [4]. Animal apoptosis is based on the centrality of caspases and a key pathway named the “canonical apoptotic pathway”, which was firstly described in *Caenorhabditis elegans* [5,6,7,8]. These mechanisms are evolutionarily conserved throughout the animal world, but they are not conserved in other eukaryotic organisms [9,10,11].

Oncogenic mutations causing cancer lead to the suppression of apoptotic cell death and enhanced cellular proliferation [12,13]. Apoptosis is one of the main animal intrinsic anticancer mechanisms. In contrast, neurodegenerative diseases occur in terminally differentiated neuronal cells and lead to their apoptotic death [14,15]. Also, the metabolism of cancer and neuron cells is very different. 

Otto Warburg, a Nobel prize winner, observed that cancer cells typically shift their metabolism toward non-mitochondrial anaerobic respiration [16]. He hypothesized that mitochondrial damage induces neoplastic transformation. Later, it turned out that, in cancer cells, a shift towards anaerobic respiration is not always observed (i.e., there are cancers in which mitochondrial respiration is very active). Assuming that the Warburg effect plays a role in neoplastic transformation, it may (i) stimulate proliferation, and (ii) inhibit cell death in tumor cells. There are many different mechanisms by which the Warburg effect stimulates cancer growth and suppresses apoptosis. Mathematical models suggest that due to the Warburg effect, cancer cells transform glucose into biomass more rapidly (which supports cellular proliferation).

In contrast to the cancer cells in which mitochondrial metabolism tends to be suppressed, the mitochondrial metabolism of neuron cells is extremely active. In this tissue, pathological aging leads to apoptotic cell death. It was hypothesized that an ‘inverse’ Warburg effect exists. According to this hypothesis, oxidative respiration stimulates pathological cell death. Perhaps not surprisingly, different studies have indicated that there is inverse comorbidity between cancer and neurodegenerative diseases [17,18,19,20,21].

To sum up, cancer and neurodegenerative diseases are caused by perturbations in apoptosis. There is unusual mitochondrial metabolism in affected tissues and according to the Warburg and inverse Warburg effects, metabolism impacts the development of diseases.

Mitochondria are also key players in apoptosis.

Phylogenetic studies have revealed the origin of mitochondrial apoptosis and eukaryotic oxidative respiration. Mitochondria are actually simplified endosymbiotic bacteria. In contrast to their host, mitochondria are able to use oxidative respiration but not anaerobic respiration. Kroemer suggested that apoptosis evolved during the domestication of mitochondria and ancestral mitochondria (protomitochondria) and, in some cases, used to kill host eukaryotic cells. However, for a long time, it was not clear why apoptotic machinery based on caspases was preserved exclusively in the unicellular ancestors of animals.

Apoptotic-like cell death is also observed in other non-animal eukaryotic organisms. Such death is also induced by the release of mitochondrial apoptotic factors. A huge controversy exists as to whether the cell death of other eukaryotes should be named ‘apoptosis’, since the key animal apoptotic factor, caspase, seems to be encoded exclusively by animal genomes. In a very recent paper [11], we performed ancestral state reconstruction of primordial mitochondrial apoptotic machinery used both by animals and other eukaryotes. This study indicates that apoptosis and apoptosis-like cell death evolved due to an evolutionary ’arms race’ conducted between ’prey’ protomitochondrion (ancestral mitochondria) and ’predator’ primitive eukaryotes. Additionally, it turns out that caspases were actually present in ancestral apoptotic machinery and ‘true’ caspase-based apoptosis appeared during mitochondrial domestication.

We came to the conclusion that our phylogenetic study suggests an apoptotic explanation of Warburg and inverse Warburg effects. Here, we present this hypothesis. Namely, we suggest that Warburg and inverse Warburg effects were already present in the ancestral state of eukaryotes. There was an antagonistic interaction between anaerobic “glycolytic” predators and aerobic protomitochondrial prey. Both types of organisms were trying to kill each other and suppress each other’s metabolism. The multiplication of aerobic organisms was causing predator cell death (apoptosis). These mechanisms were ‘frozen’ and maintained in extant organisms. As a result, there is a frozen conflict between “survival” glycolytic metabolism and ‘anaerobic’ apoptotic metabolism.

This hypothesis leads to experimentally testable predictions. The inactivation of apoptotic factors should cause perturbations in aerobic respiration in different eukaryotic organisms. Actually, in our recent, aforementioned paper, we showed such experiments using yeast. Yeast apoptotic machinery is extremely simplified in comparison to animal machinery. However, it turns out that inactivation of key apoptotic factors causes perturbations in mitochondrial metabolism.

The hypothesis presented here is also supported by other observations, for example, apoptotic factors, such as cytochrome *c* and apoptotic induction factors (AIFs), are directly involved in respiration and oncogenic mutations have been well described in mitochondrial genes and genes involved in respiration. 

The medical implications of our hypothesis are also discussed in this paper, including implications for nutrition. We also discuss the testable expectations based on our hypothesis.

## 2. Aging

Aging is one of the biggest mysteries of biology. In humans, health is most stable beyond the ages of about 50 years old [3]. In the case of older humans, age has a significant impact on health and mortality, i.e., the probability of serious illness or death increases rapidly with age. The risks for developing cardiovascular diseases, diabetes, visual impairment, dementia, and cancer dramatically increase with age [1,2,3]. In the majority of cases, age-related diseases are caused by molecular changes and molecular degeneration. There is an age-related accumulation of mutations in DNA [22,23] and protein aggregation leading to the formation of toxic protein aggregates [24,25]. Aging causes disruption in the homeostasis of whole organisms [23]. A portion of the pathological changes associated with aging are caused by dysregulation of apoptotic programmed cell death and cellular proliferation, leading to cancer and neurodegenerative diseases. Such dysregulation leads to cancer in tissues in which programmed cell death is suppressed [26,27]. There are tissues in which mitochondrial apoptotic machinery is particularly active in young organisms [28]. In contrast, in neuron cells, pathological activation of apoptotic cell death leads to neurodegenerative diseases, such as Alzheimer’s disease [14] or Parkinson’s disease [15].

## 3. Animal Apoptosis—A Form of Programmed Cell Death?

Animal apoptosis is a very particular kind of cell death [29], which was first described by Kerr in 1972 [4]. It can be easily distinguished from other kinds of cell death using morphological and biochemical features. At the initial stage of apoptosis, there is a membrane permeability transition, characterized by the breakdown of the inner mitochondrial transmembrane potential. In the next stage, chromatin condensation and nuclear fragmentation occur. Then, dying cells are fragmented into apoptotic bodies with ultrastructurally well-preserved fragments covered by membranes which are ingested by other cells. The cytoplasmic material of dying cells is isolated from the immune system by the membranes of apoptotic bodies. So, the production of apoptotic bodies is a mechanism by which organisms avoid the activation of the immune system and inflammation [29].

In classical studies performed on *C. elegans*, it was shown that animal apoptosis is a type of programmed and not accidental cell death. Adult worms have a predetermined number of cells—an adult hermaphrodite has 959 cells, and an adult male has 1031. During development, a predetermined number of cells dies (131 in case of the hermaphrodite) [30,31]. Apparently, in this case, cell death is programmed. It turns out that these cells are apoptotic [32]. It was shown later that apoptosis plays an important role in the biology of different animal organisms and medicine [29]. As mentioned previously, dysregulation of apoptosis leads to cancer and neurodegenerative diseases. However, the mechanisms of such dysregulations are opposite. In the case of cancer, there is a pathological repression of apoptosis. In the case of neurodegenerative diseases, such as Alzheimer’s disease, apoptosis is activated.

## 4. Cancer and Apoptosis

Apoptosis is a key part of the intrinsic tumor suppression mechanisms. During neoplastic transformation, malignant stresses occur, such as hypoxia, perturbation of microtubule depolymerization during the mitotic G2/M transition, and genomic instability. Such stresses induce apoptosis. Different anticancer therapies are based on the induction of apoptosis [27,29,33]. Radiotherapy [34] and cisplatin [35] induce apoptosis by damaging DNA and artificially inducing genomic instability. Taxol-based drugs cause microtubule depolymerization during the mitotic G2/M transition by stabilizing microtubules during mitosis [36,37,38].

## 5. Neurodegenerative Diseases and Apoptosis

Proteins tend to aggregate in highly organized, non-functional amyloid structures [36]. Although amyloid formation is inevitable, it is slow and subject to a kinetic barrier [36]. Apparently, amyloids are mainly homo-polymers. Different disorders, such as Parkinson’s disease, Alzheimer’s disease, spongiform encephalopathy, and type II diabetes, are caused by the formation of toxic amyloid fibrils. In the case of Parkinson’s disease, central roles are played by the formation of α-synuclein amyloid fibrils [25,39], whereby the aggregation of synuclein leads to disease [40,41,42]. The critical feature of Alzheimer’s disease is the deposition of amyloid beta (Aβ) peptide [14,43,44,45]. Both diseases lead to pathological apoptosis in neurons [14,15].

## 6. Inverse Comorbidity of Cancer and Apoptosis

It has been shown that the occurrence of both cancer and Alzheimer’s dementia increases exponentially with age [1,2,3,21]. However, different studies have shown that cancer and neurodegenerative diseases are inverse comorbidities between. It has been shown that there are low cancer rates among patients with Parkinson’s [17,19,20,46] and Alzheimer’s diseases [17,18,21]. In contrast, people with a cancer or with a history of cancer have a reduced risk of these diseases [17,18,19,20,21]. 

## 7. Warburg Hypothesis

Normal, healthy cells use oxidative mitochondrial phosphorylation for respiration. In contrast, in many cancers, a shift is observed from oxidative mitochondrial respiration towards non-oxidative respiration. This is a classical Warburg observation [16]. He claims that “Cancer cells originate from normal body cells in two phases. The first phase is the irreversible injuring of respiration. (...) The irreversible injuring of respiration is followed, as the second phase of cancer formation, by a long struggle for existence by the injured cells to maintain their structure, in which a part of the cells perish from lack of energy, while another part succeed in replacing the irretrievably lost respiration energy by fermentation energy”.

Later, different oncogenic mutations were described. It turns out that apparently, “mitochondrial damage” is not the main factor leading to cancer disease. It was shown that the overexpression of glycolytic genes, which is a hallmark of the Warburg effect, is observed in about 71% of cancers [47]. Partly, this observation could be explained by hypoxic conditions in the tumor environment. It has been shown that prolonged hypoxia causes a shift towards mitochondrial respiration [48]. However, as already mentioned in the classical papers of Warburg, usually cancer cells use mainly non-oxidative forms of respiration, even in the presence of sufficient oxygen, to support mitochondrial oxidative phosphorylation [16,49]. Different studies have indicated that Warburg effect is beneficial for cancer cells. For example, it has been shown that in human tumor cells, the Warburg effect can be reversed by replacing the embryonal isoform of pyruvate kinase expressed in cancers with its adult isoform. Such modified cancer cells have increased oxygen consumption. This reversion of the Warburg effect correlates with a reduced ability to form tumors in nude mouse xenografts [50]. Faubert and colleagues showed that the inactivation of gene AMPK (5′AMP-activated protein kinase) induces a shift towards anaerobic respiration and causes more rapid development of cancers in transgenic mice which have a predisposition for cancer formation [51]. A very recent physics paper indicates that it is likely that cancer “glycolytic” cells have higher temperature than other cells [52].

However, the Warburg effect is not an obligatory change leading to neoplastic transformation, as there are cancers in which the Warburg effect is not observed or is even reversed, i.e., cancer cells use mainly mitochondrial respiration [53].

Assuming that the Warburg effect plays a role in neoplastic transformation, it may (i) stimulate proliferation or (ii) stimulate cell death in tumor cells. The Warburg effect has a pleiotropic impact on cancer and supports its development by many different mechanisms. As a result, it is very hard to judge which of them play crucial roles. However, the majority of scholars believe that the most important factor is the first explanation. For example, in very recent seminal reviews, the second explanation was barely mentioned or not mentioned at all [54,55]. There are many mechanisms involved in the stimulation of cancer cell proliferation by the Warburg effect [54]. The Warburg effect is unexpected and puzzling, as oxidative phosphorylation is much more efficient (e.g., generates more ATP) than fermentation. Anaerobic respiration is more efficient than aerobic metabolism at incorporating nutrients into biomass. In their paper, Thomson and coworkers pointed out that “For most mammalian cells in culture, the only two molecules catabolized in appreciable quantities are glucose and glutamine.” [49]. Therefore, it is beneficial for cells to use glucose as a source of carbon instead of using it to produce ATP and CO_2_ during mitochondrial respiration. Lloyd Demetrius developed a mathematical model which indicates that the Warburg effect is more efficient at incorporating nutrients into biomass [56]. Another possible mechanism involved in the stimulation of cancer cell proliferation by the Warburg effect emerged from a recent study which showed that the synthesis of ATP is much more rapid due to anaerobic respiration [57]. In detail, it turns out that the production of lactate from glucose occurs 10–100 times faster than the complete oxidation of glucose in the mitochondria.

Other scholars point out that the Warburg effect could have a beneficial effect on the impact of cancer cells on the tumor microenvironment. It causes acidification due to elevated lactate synthesis during anaerobic respiration [54,58]. It has been shown that acidity causes increased invasiveness of cancer [58] and that lactate is a hormone that has beneficial activity for cancer. Lactate is involved in angiogenesis, immune escape, cell migration, and metastasis [59]. A recent experimental study provided indications that the Warburg effect suppresses the antitumor activity of immune T cells in tumor microenvironments. According to the suggested model, glucose is required for the proper anticancer activity of these cells [60]. Due to the Warburg effect, there is elevated glucose consumption by cancer cells and a decreased glucose level in the tumor microenvironment. A decreased glucose level inactivates the proper antitumor activity of T cells.

The second explanation is less popular, although has been considered by some scholars. However, different mechanisms of apoptosis suppression in ‘glycolytic’ cells have been described.

It has been shown many times that the inactivation of apoptosis in cancerous cells induces the Warburg effect. Tumor suppressing genes are often directly involved in the regulation of apoptosis and metabolism (see, as a review, Ref. [61]). Tumor-associated mutations or perturbations suppress the activity of apoptotic factors such as P53 [62] and PTEN [63,64,65]. Inactivation of these factors causes the Warburg effect [65,66,67]. Neoplastic transformation leads to pathological activation of oncogenic anti-apototic genes such as HIF [68,69], BCL-2 [70,71], and survivine [37]. These factors induce glycolysis [72,73,74,75].

Indeed, it has been shown many times that the Warburg effect suppresses apoptosis in cancerous cells. Glycolysis inactivates the apoptotic activity of cytochrome *c* [76]. The pro-apoptotic activity of cytochrome *c* is influenced by its redox state. Glycolysis leads to higher levels of glutathione, which reduces and inactivates the apoptotic activity of cytochrome *c*. Cancer cells have high mitochondrial membrane potential (DeltaPsim) and low expression of the K^+^ channel Kv1.5, contributing to both apoptosis resistance and the Warburg effect [77].

It was shown that the anticancer drug candidates dichloroacetate [77], metformin [78,79], SR9243 [80] and therapeutical RNAi [81] shift metabolism from glycolysis to glucose oxidation and induce apoptosis. Fasting during anticancer therapy has a similar effect [82].

In conclusion, the Warburg effect has a pleiotropic impact on cancer cells, inducing both their multiplication and the suppression of apoptosis.

## 8. The Inverse Warburg Hypothesis

In contrast to the immortal cancer cells, neuron cells are terminally differentiated. Such cells also predominantly use aerobic mitochondrial respiration. As described above, cancer and neurodegenerative diseases are inverse comorbidities [17,18,19,20,21].

These observations led to the formulation of the ‘Inverse Warburg hypothesis’. According to this hypothesis, active mitochondrial metabolism accelerates cellular aging. In agreement with the suggested model, regulation of mitochondrial respiration would compensate for the mitochondrial dysfunction that occurs during pathological aging [83,84]. In accordance with this model, it has been shown that an early marker of neuron susceptibility to Alzheimer’s disease is an increase in mtDNA and in levels of the cytochrome oxidase protein [85].

A recent paper showed that mitochondrial metabolism is regulated in opposing ways in Alzheimer’s disease and lung cancer [86]. The authors performed a bioinformatics analysis of modern transcriptomic data to check which genes are activated or repressed in both diseases and observed that genes involved in mitochondrial metabolism are regulated oppositely. This observation supports the hypothesis that mitochondrial metabolism is involved in the inverse comorbidity between these diseases.

## 9. Mitochondria Are Key Players in Oxidative Respiration and Apoptosis

As shown in previous chapters, pathological suppression of apoptosis occurs in cancer tissue in which aerobic respiration tends to be lower. In contrast, pathological activation of apoptosis during neurodegenerative diseases occurs in neurons, where oxidative respiration is particularly active. 

One can ask whether there is any connection between oxidative respiration and apoptosis. Previous studies have indicated that such connection actually exists. There is one key player common for apoptosis and oxidative respiration. This common connection is mitochondria.

Aerobic respiration takes place in the mitochondria. Indeed, mitochondria are key players in animal apoptosis and are involved in different apoptotic initiation pathways. For example, apoptosis is initiated by mitochondrial permeability transition, regulated by BCL-2 type proteins [70,87,88,89]. It induces the release of cytochrome *c* [90]. Cytochrome *c* is part of the oxidative chain and is also an apoptotic factor [85]. Released cytochrome *c* induces the proteolytic activity of caspase, which is the main animal apoptotic protease [7]. The mechanism of this activation has been well described. Cytochrome *c* is also part of a multiprotein apoptosis-activating complex called the apoptosome [7]. There are many other mitochondrial apoptotic factors, such as EndoG nuclease, OMI/HTRA proteases, apoptotic induction factors, and some caspases [29].

## 10. The Endosymbiotic Theory of Origin of Apoptosis and Oxidative Respiration

The hypothesis of the mitochondrial origin of apoptosis was first postulated by Margulis [91]. According to this theory, mitochondria evolved from bacterial ancestors (protomitochondrions) due to endosymbiosis. Mitochondria possess the machinery required for aerobic respiration. Acquiring this machinery was beneficial for the ancient eukaryotes (named protoeukaryotes). Because mitochondria are also main organelles involved in apoptosis, the mitochondrial theory of apoptotic origin was formulated firstly by Kroemer [92]. According to his theory, extant apoptotic factors are modified bacterial toxins used by protomitochondrions against their host. This theory was later tested using phylogenetic studies by Koonin and Aravind [93,94]. These studies showed that apoptotic factors actually have a bacterial origin. However, early phylogenetic studies did not explain key details. It was not clear, for example, why apoptotic machinery based on caspases was preserved exclusively in multicellular animals, but not in other eukaryotes, such as plants or fungi. In addition, the function of apoptosis based on caspases in unicellular ancestors of animals was unclear.

## 11. Apoptotic-Like Cell Death (Apoptosis?) of Non-Animal Organisms

Although genomes of non-animal organisms do not encode the key animal apoptotic factor, caspase, apoptotic-like cell death has been described in many such organisms. Whether such cell death should be named apoptosis is a controversial topic. Recently, yeast biologists decided that since it is so similar to classical apoptosis, it make no sense to use different terms [95]. Non-animal apoptosis has many cytological and biochemical hallmarks of classical animal apoptosis. It is also initiated by the release of mitochondrial “apoptotic” factors. During cell death, self-destruction of DNA is also observed. Such organisms do not use caspases, which are the main animal apoptotic proteases. However, caspase is replaced by another remotely related protease, called metacaspase. Indeed, many orthologs of several animal apoptotic factors play critical roles in apoptotic-like cell death in non-animal organisms (for example, apoptotic induction factors (AIFs), nuclease EndoG, or cytochrome *c*) [11,29,96].

The function of apoptotic like-cell death of unicellular organisms is very controversial. Some scholars claim that this type of cell death is not regulated and rather, is accidental [97]. However, there are many described examples in which apoptosis is described as a form of altruistic cell suicide [29,98,99,100].

## 12. Ancestral State Reconstruction of Apoptosis Machinery

Recently, we questioned whether animal apoptosis and apoptosis-like cell death of other eukaryotes have a common origin [11]. To answer this question, we performed ancestral state reconstruction. Using parsimony assumption, we checked which elements of apoptotic machinery were present in an ancestral state in the first eukaryotes. It turns out that this machinery was rather complex. According to our reconstruction, the ancestral eubacterial apoptotic machinery contained both caspases and metacaspases, four types of AIFs, both fungal and animal OMI/HTRA proteases, and various apoptotic DNases. Different ancient factors were lost in different clades. For example, the aforementioned caspases were lost in the majority of non-animal eukaryotes. However, it is worth mentioning that through the use of phylogenetic searches, we discovered that caspases are also encoded by the recently sequenced unicellular organism *Reticulomyxa*. Our reconstruction suggests that apoptosis evolved due to a putative evolutionary arms race between primitive eukaryotes, which were predators, and protomitochondrions, which were prey. Prey produced as many toxins as possible. These toxins were transformed into extant apoptotic factors during evolution. Different toxins were lost in different systematic groups. As a result, different organisms use different toxins/apoptotic factors. For example, animals use caspases, but other eukaryotic organisms use metacaspases. 

## 13. An Apoptotic Explanation of the Warburg and the Inverse Warburg Hypotheses

**Figure 1:**
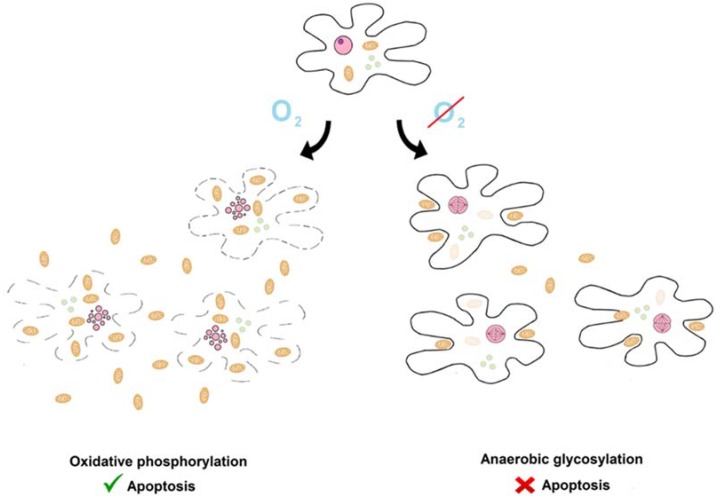
The Warburg and inverse Warburg effects in the ancestral state. There was an antagonistic relationship between prey (protomitochondrion, camel ovoids) and predators (protoeurkaryotes, black and doted lines). They killed each another. There was a dynamic equilibrium. Aerobic conditions caused shift of balance towards protomitochondrions and protoeukaryotes were killed (inverse Warburg effect). In contrast, under anaerobic conditions, the struggle was won by protoeukarytes. Other graphical elements: pink ovals—nucleus or fragmented nucleus, greenish circles—vesicles.

Here, we present the hypothesis that the impact of cellular metabolism on apoptosis described by Warburg and inverse Warburg hypotheses already existed in the ancestral state (see Figure 1). In this state, the activity of the aerobic respiration and apoptotic systems was linearly proportional to the number of living protomitochondrions. Protomitochondrions multiplied under conditions supporting oxidative respiration and killed protoeukaryotes. This phenomenon was a primitive form of the Warburg phenomenon.In contrast, under conditions supporting glycolytic respiration, primitive eukaryotes multiplied rapidly and killed protomitochondrions. This was the primitive inverse Warburg phenomenon.

This ancient mechanism was somehow maintained over evolutionary processes and is present in current human cells.

## 14. The Warburg and the Inverse Warburg Hypotheses in Yeast

Assuming that principles described by the Warburg and the inverse Warburg hypotheses have an endosymbiotic origin, such principles should also be observed in non-animal eukaryotes. Actually, recent studies suggest that the Warburg [11] and inverse Warburg hypotheses are observed in yeast [101].

We showed that cancer-like inactivation of apoptotic factors in yeast also causes perturbations in aerobic respiration [11]. We investigated the well-described apoptotic factors NDI1, a mitochondrial apoptotic induction factor (AIF); NUC1, a mitochondrial apoptotic DNase ENDOG; MCA1, a cytoplasmic metacaspase; and NMA111, a cytoplasmic apoptotic protease, using yeast *Saccharomyces cerevisiae* W303. Deletion of these factors causes cancer-like suppression of apoptotic activity. It turns out that such deletions are beneficial under anaerobic conditions. During co-cultivation experiments performed under anaerobic conditions, wild-type cells gradually lost competitions with mutants. In contrast, under aerobic conditions, all of the studied mutants lost competitions with wild-type cells. To verify how the studied deletions affect the ability to perform mitochondrial respiration, we examined the growth of the mutants and wild-type cells on a medium containing glycerol as a source of carbon. The growth of yeast in this medium requires mitochondrial respiration. It turns out that ‘apoptotic’ mutants were not able to grow on this medium.

In conclusion, the results presented above indicate that in yeast, cancer-like inactivation of apoptotic machinery leads to remodeling of metabolism and a shift towards anaerobic respiration. 

Recent studies also indicate that, in yeast, as postulated by the ‘inverse Warburg hypothesis’, the activation of apoptotic activity occurs in cells which use mitochondrial respiration. Indeed, some important details describing this process have been revealed. It was shown that the activation of mitochondrial respiration contributes to the activation of RAS-dependent apoptosis. RAS protein accumulates mainly at the plasma membrane and in the nucleus during growth on medium containing glucose [101,102]. In contrast, it localizes mainly in the mitochondria in wild-type glucose-starved cells. However, it moves from the mitochondria to the nucleus after the addition of glucose to the medium. Also, the deletion of hexokinases—key enzymes of anaerobic respiration—causes mitochondrial localization of RAS small GTPase. Additionally, it has been shown that the activation of apoptosis by acetic acid causes translocation of RAS from the plasma membrane to the mitochondria. Furthermore, it was shown that the activation of apoptosis induced by acetic acid and H_2_O_2_ is stronger in the above mentioned deletion mutants with inactivated hexokinases [101,102]. It also turns out that RAS-dependent apoptosis is metacaspase-independent and that AIF1 is involved in the apoptotic pathway induced by RAS [102].

The apoptotic explanation of the inverse Warburg hypothesis presented here also predicts that the mechanism of apoptosis activation by neurotoxic aggregates is evolutionary conserved and cells could be rescued by inactivation of mitochondrial metabolism.

Actually, recent studies with yeast models confirmed this expectation in the case of Parkinson’s disease. As already mentioned, Parkinson’s disease is caused by the aggregation of α-synuclein [42]. Heterologous expression of this protein in yeast is toxic for aging cells [103]. It turns out that apoptosis induced by toxic aggregates requires endonuclease G (ENDOG). Similar observations were made in *C. elegans* and flies. In *C. elegans*, heterologous expression of human α-synuclein is neurotoxic, but deletion of EndoG protects these cells. In flies, heterologous expression of human α-synuclein is also neurotoxic, and depletion of EndoG by RNA interference protects neuron cells as well [40]. It was also shown that in yeast, functional mitochondria are required for synuclein toxicity. The abrogation of mitochondrial DNA (rho0 type mutation) protects cells against the toxic activity of α-synuclein aggregates [103].

In the case of Alzheimer’s disease, the situation is less obvious. During the progression of the disease, toxicity is caused by the aggregation of amyloid beta peptide. In humans, toxic forms of this peptide are generated by proteolytic cleavage of APP, the transmembrane amyloid precursor protein, during processing in the secretory pathway. The expression of amyloid human beta peptide is toxic for yeast when it is directed to secretion by fusion to an ER targeting signal to the N-terminus. It has been shown that such toxic peptides have an impact on mitochondria metabolism [104]. A very recent study showed that toxicity caused by human amyloid beta peptide affects mitochondria. Such mitochondria have structures typical of aged yeast (they are more fragmented). Indeed, there is an increase in the production of reactive oxygen species (ROS) in cells accumulating amyloid beta. It is important to note that the production of ROS is a typical hallmark of apoptosis [105].

In conclusion, the apoptotic mechanisms involved in Warburg and inverse Warburg are very old. In both yeast and animals, the inactivation of apoptosis leads to the repression of aerobic mitochondrial respiration. In contrast, the activation of mitochondrial metabolism plays a role in the activation of apoptosis. Indeed, the expression of human neurotoxic proteins has an identical impact in both types of organisms and activates the EndoG-dependent apoptotic pathway.A comparison of the apoptotic traits of animals (humans) and yeast are shown in Table 1.

## 15. Medical Implications of Our Observations

The hypotheses presented here have important medical implications. As already mentioned, epidemiological studies suggest that general aging is a main risk factor in the development of cancer and neurodegenerative diseases. Recent studies indicate that a general mechanism of aging exists and that this process is regulated to some extent. On the molecular level, there are at least three different biochemical pathways that control aging: insulin/insulin-like growth factor 1 (IGF-1), tuberous sclerosis complex (TSC), mammalian target of rapamycin (mTOR), and the sirtuins [106]. Caloric restriction, exercise, and proper diet have beneficial impacts on aging and the development of cancer and Alzheimer’s disease (AD). Experiments with animals have shown that caloric restriction reduces the risk of cancer and neurodegeneration [107]. It has been shown that adherence to a Mediterranean-type diet is associated with a reduced risk of AD [108]. Similar observations were made in the case of cancer [109,110]. Physical activity also reduces the risk of developing these diseases [111,112].

However, there is an inverse comorbidity between these diseases. Our hypothesis suggests that anticancer treatment may accelerate neuronal aging, and vice versa, factors inhibiting neuronal aging may stimulate neoplastic transformation. It has been shown that estrogen replacement therapy increases the probability of developing cancer [113,114] and decreases the probability of developing dementia [115]. We expect that there will be more similar cases discovered in the future.

## 16. Conclusions

In this paper, we presented an apoptotic and endosymbiotic explanation of the Warburg and the inverse Warburg hypotheses. Namely, the bacterial ancestors of extant mitochondria—protomitochondrions—produced toxins and performed oxidative respiration. The production of toxins and activity of oxidative respiration was approximately proportional to the number of protomitochondrions. This ancestral correlation between the activity of mitochondrial metabolism and apoptotic activity was maintained in yeast and animals. 

The aforementioned hypothesis led to the following testable predictions:

- The induction of apoptosis causes a metabolic shift towards aerobic respiration;
- The stimulation of aerobic respiration induces apoptosis;
- The suppression of apoptosis causes a metabolic shift towards anaerobic respiration;
- The suppression of aerobic respiration inhibits apoptosis.

As described above, these predictions have been confirmed by many experimental studies including our very recent paper. Indeed, it is expected that the ancestral correlation between activity of mitochondrial metabolism and apoptotic activity described here is maintained throughout the eukaryotic world. If this is the case, this correlation would be observed also in plants, green algae, and unicellular protozoa, in which apoptosis/apoptosis-like cell death has been well described [29,96].

In conclusion, evolutionary apoptotic history has a strong impact on extant eukaryotic organisms. 

## Figures and Tables

**Table 1:** Human and yeast apoptosis mechanisms. The table indicates that the majority of these apoptotic traits appeared before human/fungi diversification (probably even before the origin of eukaryotes).

Mechanism of Apoptosis	*S. cerevisiae*	Homo Sapiens
Caspase	−	+
Metacaspase	+	−
Cytochrome *c* induced apoptosis	+	+
Mitochondrial permeability transition	+	+
EndoG	+	+
Parkinson-like activation of EndoG apoptotic pathway by α-synuclein aggregates	+	+
AIFs (Apoptotic Induction Factors)	+	+
OMI/HTRA apoptotic protease	+	+
Suppression of apoptotic activity causes co-suppression of aerobic respiration (Warburg effect)	+	+
Aerobic respiration stimulates apoptotic activity (inverse Warburg effect)	+	+

## References

[1] Alzheimer’s Association (2016). 2016 Alzheimer’s disease facts and figures. Alzheimers Dement..

[2] Alzheimer’s Association (2015). 2015 Alzheimer’s disease facts and figures. Alzheimers Dement..

[3] Heron M.P. (2018). Deaths: Leading causes for 2016. Natl. Vital. Stat. Rep..

[4] Kerr J.F., Wyllie A.H., Currie A.R. (1972). Apoptosis: A basic biological phenomenon with wide-ranging implications in tissue kinetics. Br. J. Cancer.

[5] Hengartner M.O., Horvitz H.R. (1994). *C. elegans* cell survival gene ced-9 encodes a functional homolog of the mammalian proto-oncogene bcl-2. Cell.

[6] Yuan J., Shaham S., Ledoux S., Ellis H.M., Horvitz H.R. (1993). The, *C. elegans* cell death gene ced-3 encodes a protein similar to mammalian interleukin-1 beta-converting enzyme. Cell.

[7] Qi S., Pang Y., Hu Q., Liu Q., Li H., Zhou Y., He T., Liang Q., Liu Y., Yuan X. (2010). Crystal structure of the *Caenorhabditis elegans* apoptosome reveals an octameric assembly of CED-4. Cell.

[8] Yan N., Chai J., Lee E.S., Gu L., Liu Q., He J., Wu J.W., Kokel D., Li H., Hao Q. (2005). Structure of the CED-4-CED-9 complex provides insights into programmed cell death in *Caenorhabditis elegans*. Nature.

[9] Sakamaki K., Imai K., Tomii K., Miller D.J. (2015). Evolutionary analyses of caspase-8 and its paralogs: Deep origins of the apoptotic signaling pathways. Bioessays.

[10] Green D.R., Fitzgerald P. (2016). Just So Stories about the Evolution of Apoptosis. Curr. Biol..

[11] Klim J., Gładki A., Kucharczyk R., Zielenkiewicz U., Kaczanowski S. (2018). Ancestral State Reconstruction of the Apoptosis Machinery in the Common Ancestor of Eukaryotes. G3 (Bethesda).

[12] Evan G.I., Vousden K.H. (2001). Proliferation, cell cycle and apoptosis in cancer. Nature.

[13] Lowe S.W., Cepero E., Evan G. (2004). Intrinsic tumour suppression. Nature.

[14] LaFerla F.M., Tinkle B.T., Bieberich C.J., Haudenschild C.C., Jay G. (1995). The Alzheimer’s A beta peptide induces neurodegeneration and apoptotic cell death in transgenic mice. Nat. Genet..

[15] Mochizuki H., Goto K., Mori H., Mizuno Y. (1996). Histochemical detection of apoptosis in Parkinson’s disease. J. Neurol. Sci..

[16] Warburg O. (1956). On the origin of cancer cells. Science.

[17] Tabarés-Seisdedos R., Dumont N., Baudot A., Valderas J.M., Climent J., Valencia A., Crespo-Facorro B., Vieta E., Gómez-Beneyto M., Martínez S. (2011). No paradox, no progress: Inverse cancer comorbidity in people with other complex diseases. Lancet Oncol..

[18] Driver J.A., Beiser A., Au R., Kreger B.E., Splansky G.L., Kurth T., Kiel D.P., Lu K.P., Seshadri S., Wolf P.A. (2012). Inverse association between cancer and Alzheimer’s disease: Results from the Framingham Heart Study. BMJ.

[19] Jansson B., Jankovic J. (1985). Low cancer rates among patients with Parkinson’s disease. Ann. Neurol..

[20] West A.B., Dawson V.L., Dawson T.M. (2005). To die or grow: Parkinson’s disease and cancer. Trends Neurosci..

[21] Musicco M., Adorni F., Di Santo S., Prinelli F., Pettenati C., Caltagirone C., Palmer K., Russo A. (2013). Inverse occurrence of cancer and Alzheimer disease: A population-based incidence study. Neurology.

[22] Lodato M.A., Rodin R.E., Bohrson C.L., Coulter M.E., Barton A.R., Kwon M., Sherman M.A., Vitzthum C.M., Luquette L.J., Yandava C.N. (2018). Aging and neurodegeneration are associated with increased mutations in single human neurons. Science.

[23] Luu J., Palczewski K. (2018). Human aging and disease: Lessons from age-related macular degeneration. Proc. Natl. Acad. Sci. USA.

[24] David D.C., Ollikainen N., Trinidad J.C., Cary M.P., Burlingame A.L., Kenyon C. (2010). Widespread protein aggregation as an inherent part of aging in *C. elegans*. PLoS Biol..

[25] Chiti F., Dobson C.M. (2017). Protein Misfolding, Amyloid Formation, and Human Disease: A Summary of Progress Over the Last Decade. Ann. Rev. Biochem..

[26] Reed J.C. (1999). Dysregulation of apoptosis in cancer. J. Clin. Oncol..

[27] Pistritto G., Trisciuoglio D., Ceci C., Garufi A., D’Orazi G. (2016). Apoptosis as anticancer mechanism: Function and dysfunction of its modulators and targeted therapeutic strategies. Aging.

[28] Sarosiek K.A., Fraser C., Muthalagu N., Bhola P.D., Chang W., McBrayer S.K., Cantlon A., Fisch S., Golomb-Mello G., Ryan J.A. (2017). Developmental Regulation of Mitochondrial Apoptosis by c-Myc Governs Age- and Tissue-Specific Sensitivity to Cancer Therapeutics. Cancer Cell.

[29] Kaczanowski S. (2016). Apoptosis: Its origin, history, maintenance and the medical implications for cancer and aging. Phys. Biol..

[30] Sulston J.E., Horvitz H.R. (1977). Post-embryonic cell lineages of the nematode, *Caenorhabditis elegans*. Dev. Biol..

[31] Sulston J.E., Schierenberg E., White J.G., Thomson J.N. (1983). The embryonic cell lineage of the nematode *Caenorhabditis elegans*. Dev. Biol..

[32] Robertson A.M., Thomson J. (1982). Morphology of programmed cell death in the ventral nerve cord of *Caenorhabditis elegans* larvae. Development.

[33] Visconti R., Della Monica R., Grieco D. (2016). Cell cycle checkpoint in cancer: A therapeutically targetable double-edged sword. J. Exp. Clin. Cancer Res..

[34] Bao S., Wu Q., McLendon R.E., Hao Y., Shi Q., Hjelmeland A.B., Dewhirst M.W., Bigner D.D., Rich J.N. (2006). Glioma stem cells promote radioresistance by preferential activation of the DNA damage response. Nature.

[35] Puig P.E., Guilly M.N., Bouchot A., Droin N., Cathelin D., Bouyer F., Favier L., Ghiringhelli F., Kroemer G., Solary E. (2008). Tumor cells can escape DNA-damaging cisplatin through DNA endoreduplication and reversible polyploidy. Cell Biol. Int..

[36] Bacus S.S., Gudkov A.V., Lowe M., Lyass L., Yung Y., Komarov A.P., Keyomarsi K., Yarden Y., Seger R. (2001). Taxol-induced apoptosis depends on MAP kinase pathways (ERK and p38) and is independent of p53. Oncogene.

[37] Li F., Ambrosini G., Chu E.Y., Plescia J., Tognin S., Marchisio P.C., Altieri D.C. (1998). Control of apoptosis and mitotic spindle checkpoint by survivin. Nature.

[38] Zhou M., Zhao Y., Ding Y., Liu H., Liu Z., Fodstad O., Riker A.I., Kamarajugadda S., Lu J., Owen L.B. (2010). Warburg effect in chemosensitivity: Targeting lactate dehydrogenase-A re-sensitizes taxol-resistant cancer cells to taxol. Mol. Cancer.

[39] Dobson C.M. (2001). The structural basis of protein folding and its links with human disease. Philos. Trans. R. Soc. Lond. B Biol. Sci..

[40] Büttner S., Habernig L., Broeskamp F., Ruli D., Vögtle F.N., Vlachos M., Macchi F., Küttner V., Carmona-Gutierrez D., Eisenberg T. (2013). Endonuclease G mediates α-synuclein cytotoxicity during Parkinson’s disease. EMBO J..

[41] Flagmeier P., Meisl G., Vendruscolo M., Knowles T.P., Dobson C.M., Buell A.K., Galvagnion C. (2016). Mutations associated with familial Parkinson’s disease alter the initiation and amplification steps of α-synuclein aggregation. Proc. Natl. Acad. Sci. USA.

[42] Spillantini M.G., Crowther R.A., Jakes R., Hasegawa M., Goedert M. (1998). Alpha-Synuclein in filamentous inclusions of Lewy bodies from Parkinson’s disease and dementia with lewy bodies. Proc. Natl. Acad. Sci. USA.

[43] Selkoe D.J., Hardy J. (2016). The amyloid hypothesis of Alzheimer’s disease at 25 years. EMBO Mol. Med..

[44] Glenner G.G., Wong C.W. (1984). Alzheimer’s disease: Initial report of the purification and characterization of a novel cerebrovascular amyloid protein. Biochem. Biophys. Res. Commun..

[45] Vassar R., Bennett B.D., Babu-Khan S., Kahn S., Mendiaz E.A., Denis P., Teplow D.B., Ross S., Amarante P., Loeloff R. (1999). Beta-secretase cleavage of Alzheimer’s amyloid precursor protein by the transmembrane aspartic protease BACE. Science.

[46] Ong E.L., Goldacre R., Goldacre M. (2014). Differential risks of cancer types in people with Parkinson’s disease: A national record-linkage study. Eur. J. Cancer.

[47] Altenberg B., Greulich K.O. (2004). Genes of glycolysis are ubiquitously overexpressed in 24 cancer classes. Genomics.

[48] Rodríguez-Enríquez S., Carreño-Fuentes L., Gallardo-Pérez J.C., Saavedra E., Quezada H., Vega A., Marín-Hernández A., Olín-Sandoval V., Torres-Márquez M.E., Moreno-Sánchez R. (2010). Oxidative phosphorylation is impaired by prolonged hypoxia in breast and possibly in cervix carcinoma. Int. J. Biochem. Cell Biol..

[49] Vander Heiden M.G., Cantley L.C., Thompson C.B. (2009). Understanding the Warburg effect: The metabolic requirements of cell proliferation. Science.

[50] Christofk H.R., Vander Heiden M.G., Harris M.H., Ramanathan A., Gerszten R.E., Wei R., Fleming M.D., Schreiber S.L., Cantley L.C. (2008). The M2 splice isoform of pyruvate kinase is important for cancer metabolism and tumour growth. Nature.

[51] Faubert B., Boily G., Izreig S., Griss T., Samborska B., Dong Z., Dupuy F., Chambers C., Fuerth B.J., Viollet B. (2013). AMPK is a negative regulator of the Warburg effect and suppresses tumor growth in vivo. Cell Metab..

[52] Zivieri R., Pacini N., Finocchio G., Carpentieri M. (2017). Rate of entropy model for irreversible processes in living systems. Sci. Rep..

[53] Zheng J. (2012). Energy metabolism of cancer: Glycolysis versus oxidative phosphorylation (Review). Oncol. Lett..

[54] Liberti M.V., Locasale J.W. (2016). The Warburg Effect: How Does it Benefit Cancer Cells?. Trends Biochem. Sci..

[55] Chen X., Qian Y., Wu S. (2015). The Warburg effect: Evolving interpretations of an established concept. Free Radic. Biol. Med..

[56] Demetrius L.A., Coy J.F., Tuszynski J.A. (2010). Cancer proliferation and therapy: The Warburg effect and quantum metabolism. Theor. Biol. Med. Model.

[57] Shestov A.A., Liu X., Ser Z., Cluntun A.A., Hung Y.P., Huang L., Kim D., Le A., Yellen G., Albeck J.G. (2014). Quantitative determinants of aerobic glycolysis identify flux through the enzyme GAPDH as a limiting step. Elife.

[58] Estrella V., Chen T., Lloyd M., Wojtkowiak J., Cornnell H.H., Ibrahim-Hashim A., Bailey K., Balagurunathan Y., Rothberg J.M., Sloane B.F. (2013). Acidity generated by the tumor microenvironment drives local invasion. Cancer Res..

[59] San-Millán I., Brooks G.A. (2017). Reexamining cancer metabolism: Lactate production for carcinogenesis could be the purpose and explanation of the Warburg Effect. Carcinogenesis.

[60] Chang C.H., Qiu J., O’Sullivan D., Buck M.D., Noguchi T., Curtis J.D., Chen Q., Gindin M., Gubin M.M., van der Windt G.J. (2015). Metabolic Competition in the Tumor Microenvironment Is a Driver of Cancer Progression. Cell.

[61] Levine A.J., Puzio-Kuter A.M. (2010). The control of the metabolic switch in cancers by oncogenes and tumor suppressor genes. Science.

[62] Attardi L.D. (2005). The role of p53-mediated apoptosis as a crucial anti-tumor response to genomic instability: Lessons from mouse models. Mutat. Res..

[63] Weng L., Brown J., Eng C. (2001). PTEN induces apoptosis and cell cycle arrest through phosphoinositol-3-kinase/Akt-dependent and -independent pathways. Hum. Mol. Genet..

[64] Choi Y., Zhang J., Murga C., Yu H., Koller E., Monia B.P., Gutkind J.S., Li W. (2002). PTEN, but not SHIP and SHIP2, suppresses the PI3K/Akt pathway and induces growth inhibition and apoptosis of myeloma cells. Oncogene.

[65] Cordero-Espinoza L., Hagen T. (2013). Increased concentrations of fructose 2,6-bisphosphate contribute to the Warburg effect in phosphatase and tensin homolog (PTEN)-deficient cells. J. Biol. Chem..

[66] Itahana Y., Itahana K. (2018). Emerging Roles of p53 Family Members in Glucose Metabolism. Int. J. Mol. Sci..

[67] Eriksson M., Ambroise G., Ouchida A.T., Lima Queiroz A., Smith D., Gimenez-Cassina A., Iwanicki M.P., Muller P.A., Norberg E., Vakifahmetoglu-Norberg H. (2017). Effect of Mutant p53 Proteins on Glycolysis and Mitochondrial Metabolism. Mol. Cell. Biol..

[68] Sasabe E., Tatemoto Y., Li D., Yamamoto T., Osaki T. (2005). Mechanism of HIF-1alpha-dependent suppression of hypoxia-induced apoptosis in squamous cell carcinoma cells. Cancer Sci..

[69] Mimeault M., Batra S.K. (2013). Hypoxia-inducing factors as master regulators of stemness properties and altered metabolism of cancer- and metastasis-initiating cells. J. Cell. Mol. Med..

[70] Delbridge A.R., Strasser A. (2015). The BCL-2 protein family, BH3-mimetics and cancer therapy. Cell Death Differ..

[71] Sinicrope F.A., Ruan S.B., Cleary K.R., Stephens L.C., Lee J.J., Levin B. (1995). Bcl-2 and p53 oncoprotein expression during colorectal tumorigenesis. Cancer Res..

[72] Hagenbuchner J., Kiechl-Kohlendorfer U., Obexer P., Ausserlechner M.J. (2016). BIRC5/Survivin as a target for glycolysis inhibition in high-stage neuroblastoma. Oncogene.

[73] Hagenbuchner J., Kuznetsov A.V., Obexer P., Ausserlechner M.J. (2013). BIRC5/Survivin enhances aerobic glycolysis and drug resistance by altered regulation of the mitochondrial fusion/fission machinery. Oncogene.

[74] Mahmood B., Wilson J., Ahmad M., Olino P., King J., Dejean L. (2015). Bcl-2 Overexpression Stimulates Glycolysis and Lactic Fermentation in a Bax-Dependent Fashion. Biophys. J..

[75] Semenza G.L. (2007). HIF-1 mediates the Warburg effect in clear cell renal carcinoma. J. Bioenerg. Biomembr..

[76] Vaughn A.E., Deshmukh M. (2008). Glucose metabolism inhibits apoptosis in neurons and cancer cells by redox inactivation of cytochrome *c*. Nat. Cell Biol..

[77] Bonnet S., Archer S.L., Allalunis-Turner J., Haromy A., Beaulieu C., Thompson R., Lee C.T., Lopaschuk G.D., Puttagunta L., Harry G. (2007). A mitochondria-K+ channel axis is suppressed in cancer and its normalization promotes apoptosis and inhibits cancer growth. Cancer Cell.

[78] Jia Y., Ma Z., Liu X., Zhou W., He S., Xu X., Ren G., Xu G., Tian K. (2015). Metformin prevents DMH-induced colorectal cancer in diabetic rats by reversing the warburg effect. Cancer Med..

[79] Harada K., Ferdous T., Harada T., Ueyama Y. (2016). Metformin in combination with 5-fluorouracil suppresses tumor growth by inhibiting the Warburg effect in human oral squamous cell carcinoma. Int. J. Oncol..

[80] Flaveny C.A., Griffett K., El-Gendy B.D., Kazantzis M., Sengupta M., Amelio A.L., Chatterjee A., Walker J., Solt L.A., Kamenecka T.M. (2015). Broad Anti-tumor Activity of a Small Molecule that Selectively Targets the Warburg Effect and Lipogenesis. Cancer Cell.

[81] Takai T., Yoshikawa Y., Inamoto T., Minami K., Taniguchi K., Sugito N., Kuranaga Y., Shinohara H., Kumazaki M., Tsujino T. (2017). A Novel Combination RNAi toward Warburg Effect by Replacement with miR-145 and Silencing of PTBP1 Induces Apoptotic Cell Death in Bladder Cancer Cells. Int. J. Mol. Sci..

[82] Bianchi G., Martella R., Ravera S., Marini C., Capitanio S., Orengo A., Emionite L., Lavarello C., Amaro A., Petretto A. (2015). Fasting induces anti-Warburg effect that increases respiration but reduces ATP-synthesis to promote apoptosis in colon cancer models. Oncotarget.

[83] Demetrius L.A., Magistretti P.J., Pellerin L. (2014). Alzheimer’s disease: The amyloid hypothesis and the Inverse Warburg effect. Front Physiol..

[84] Demetrius L.A., Simon D.K. (2013). The inverse association of cancer and Alzheimer’s: A bioenergetic mechanism. J. R. Soc. Interface.

[85] Zhu X., Perry G., Moreira P.I., Aliev G., Cash A.D., Hirai K., Smith M.A. (2006). Mitochondrial abnormalities and oxidative imbalance in Alzheimer disease. J. Alzheimers Dis..

[86] Ibáñez K., Boullosa C., Tabarés-Seisdedos R., Baudot A., Valencia A. (2014). Molecular evidence for the inverse comorbidity between central nervous system disorders and cancers detected by transcriptomic meta-analyses. PLoS Genet..

[87] Marchetti P., Castedo M., Susin S.A., Zamzami N., Hirsch T., Macho A., Haeffner A., Hirsch F., Geuskens M., Kroemer G. (1996). Mitochondrial permeability transition is a central coordinating event of apoptosis. J. Exp. Med..

[88] Kroemer G., Reed J. (2000). Mitochondrial control of cell death. Nat. Med..

[89] Petit P., Susin S., Zamzami N., Mignotte B., Kroemer G. (1996). Mitochondria and programmed cell death: Back to the future. FEBS Lett..

[90] Kluck R., Bossy-Wetzel E., Green D., Newmeyer D. (1997). The release of cytochrome *c* from mitochondria: A primary site for Bcl-2 regulation of apoptosis. Science.

[91] Margulis L. (1993). Symbiosis in Cell Evolution.

[92] Kroemer G. (1997). Mitochondrial implication in apoptosis. Towards an endosymbiont hypothesis of apoptosis evolution. Cell Death Differ..

[93] Koonin E.V., Aravind L. (2002). Origin and evolution of eukaryotic apoptosis: The bacterial connection. Cell Death Differ..

[94] Aravind L., Dixit V.M., Koonin E.V. (2001). Apoptotic molecular machinery: Vastly increased complexity in vertebrates revealed by genome comparisons. Science.

[95] Carmona-Gutierrez D., Bauer M.A., Zimmermann A., Aguilera A., Austriaco N., Ayscough K., Balzan R., Bar-Nun S., Barrientos A., Belenky P. (2018). Guidelines and recommendations on yeast cell death nomenclature. Microbiol. Cell.

[96] Kaczanowski S., Sajid M., Reece S.E. (2011). Evolution of apoptosis-like programmed cell death in unicellular protozoan parasites. Parasit. Vectors.

[97] Proto W.R., Coombs G.H., Mottram J.C. (2013). Cell death in parasitic protozoa: Regulated or incidental?. Nat. Rev. Microbiol..

[98] Taylor-Brown E., Hurd H. (2013). The first suicides: A legacy inherited by parasitic protozoans from prokaryote ancestors. Parasit. Vectors.

[99] Duszenko M., Figarella K., Macleod E., Welburn S. (2006). Death of a trypanosome: A selfish altruism. Trends Parasitol..

[100] Durand P.M., Choudhury R., Rashidi A., Michod R.E. (2014). Programmed death in a unicellular organism has species-specific fitness effects. Biol. Lett..

[101] Amigoni L., Frigerio G., Martegani E., Colombo S. (2016). Involvement of Aif1 in apoptosis triggered by lack of Hxk2 in the yeast Saccharomyces cerevisiae. FEMS Yeast Res..

[102] Amigoni L., Martegani E., Colombo S. (2013). Lack of HXK2 induces localization of active Ras in mitochondria and triggers apoptosis in the yeast Saccharomyces cerevisiae. Oxid. Med. Cell. Longev..

[103] Büttner S., Bitto A., Ring J., Augsten M., Zabrocki P., Eisenberg T., Jungwirth H., Hutter S., Carmona-Gutierrez D., Kroemer G. (2008). Functional mitochondria are required for alpha-synuclein toxicity in aging yeast. J. Biol. Chem..

[104] Treusch S., Hamamichi S., Goodman J.L., Matlack K.E., Chung C.Y., Baru V., Shulman J.M., Parrado A., Bevis B.J., Valastyan J.S. (2011). Functional links between Aβ toxicity, endocytic trafficking, and Alzheimer’s disease risk factors in yeast. Science.

[105] Chen X., Bisschops M.M.M., Agarwal N.R., Ji B., Shanmugavel K.P., Petranovic D. (2017). Interplay of Energetics and ER Stress Exacerbates Alzheimer’s Amyloid-β (Aβ) Toxicity in Yeast. Front. Mol. Neurosci..

[106] Hubbard B.P., Sinclair D.A. (2014). Small molecule SIRT1 activators for the treatment of aging and age-related diseases. Trends Pharmacol. Sci..

[107] Fontana L., Partridge L., Longo V. (2010). Extending healthy life span--from yeast to humans. Science.

[108] Bhushan A., Fondell E., Ascherio A., Yuan C., Grodstein F., Willett W. (2018). Adherence to Mediterranean diet and subjective cognitive function in men. Eur. J. Epidemiol..

[109] Schwingshackl L., Schwedhelm C., Galbete C., Hoffmann G. (2017). Adherence to Mediterranean Diet and Risk of Cancer: An Updated Systematic Review and Meta-Analysis. Nutrients.

[110] Schwingshackl L., Hoffmann G. (2016). Does a Mediterranean-Type Diet Reduce Cancer Risk?. Curr. Nutr. Rep..

[111] Moore S.C., Lee I.M., Weiderpass E., Campbell P.T., Sampson J.N., Kitahara C.M., Keadle S.K., Arem H., Berrington de Gonzalez A., Hartge P. (2016). Association of Leisure-Time Physical Activity With Risk of 26 Types of Cancer in 1.44 Million Adults. JAMA Int. Med..

[112] Tolppanen A.M., Solomon A., Kulmala J., Kåreholt I., Ngandu T., Rusanen M., Laatikainen T., Soininen H., Kivipelto M. (2015). Leisure-time physical activity from mid- to late life, body mass index, and risk of dementia. Alzheimers Dement..

[113] Schairer C., Lubin J., Troisi R., Sturgeon S., Brinton L., Hoover R. (2000). Menopausal estrogen and estrogen-progestin replacement therapy and breast cancer risk. JAMA.

[114] Pike M.C., Spicer D.V., Dahmoush L., Press M.F. (1993). Estrogens progestogens normal breast cell proliferation and breast cancer risk. Epidemiol. Rev..

[115] Paganini-Hill A., Henderson V.W. (1996). Estrogen replacement therapy and risk of Alzheimer disease. Arch. Int. Med..

